# Plant biomass and seed production of the legumes *Aeschynomeme histrix* and *Stylosanthes hamata* and the potential of endozoochory by cattle and sheep in semi-arid native pastures

**DOI:** 10.1016/j.heliyon.2023.e18202

**Published:** 2023-07-12

**Authors:** Lassina Sanou, Souleymane Ouédraogo, Patrice Savadogo, Jérôme Bindelle, Chantal Yvette Kabore-Zoungrana

**Affiliations:** a Centre National de la Recherche Scientifique et Technologique, INERA, Département Environnement et Forêts, BP 7047, Ouagadougou 03, Burkina Faso; b Centre National de la Recherche Scientifique et Technologique, INERA, Gestion des Ressources Naturelles et Systèmes de Production (GRN/SP), BP 7047, Ouagadougou 03, Burkina Faso; c University of Liège, Gembloux Agro-Bio Tech, Animal Science Unit, Passage des 7 Déportés, 2, 5030 Gembloux, Belgium; d Université Nazi Boni, Institut du Développement Rural, BP 1091 Bobo-Dioulasso, Burkina Faso

**Keywords:** Experimental endozoochory, Legume, Seed dispersal, Seed survival, Improved pastures, West Africa

## Abstract

Endozoochory is a substantial vector for seed dispersal and plays an important role in vegetation dynamics, mainly in colonisation processes through seed input to the vegetation and soil seed bank. We investigated the endozoochorous seed input by cattle and sheep on a pasture located in the western region of Burkina Faso. Through germination experiments, we assessed viable seed content of the dung of these grazing animals to estimate their suitability and efficiency for seed dispersal of fodder legumes. Cattle and sheep were daily fed seeds of *Sthylosanthes hamata* and *Aeschynomene histrix,* mixed with cotton seed cake. Faeces containing seeds of both legumes were collected 24 h after feeding. One part of faeces samples was spread in buckets of soil for direct germination in the greenhouse to evaluate germinating seed content. To improve pastures, a randomized completed design with 6 replications was conducted with both legumes and phosphorus fertilization (0 and 100 kg/ha of P_2_O_5_) and year as experimental factors. Recovery of *A. histrix* seeds was better than that of *S. hamata* with cattle (18 and 9%, respectively) compared to sheep. Seed recovered from faeces had higher germination with sheep than cattle. Thus, *S. hamata* seed recovered from faeces germinated well (12 and 45% with cattle and sheep, respectively, than fresh seeds used as control. However, *A. histrix*'s seeds recovered from faeces germinated less than control (P < 0.001). The findings confirmed that ruminants could be used for targeted legume seed dispersal in natural pastures. *A. histrix* and *S. hamata* have high potential for plant biomass and seed production when phosphorus is applied. Seed ingestion by ruminants should be undertaken for improving natural pastures in semi-arid zones as lower cost practice.

## 1 Introduction

Rearing of livestock are an important economic and food security activity in many parts of the world. Natural pastures form the main source for livestock feed, thus ruminant animals depend on natural pastures and crop residues for their nutrient requirements [1]. Tropical pastures are dominated by grasses that suffer from N-deficiency, particularly during the dry season, which negatively affects their utilisation by livestock. The scope for increasing profitability of grazing animals depends on pasture performance, particularly in Sub-Saharan Africa where fodder shortage is the most important constraint to livestock production [2]. Introduction and the use of legumes are considered to be an essential part of animal husbandry [2]. The biomass production of herbaceous plants in natural pastures, which is the main fodder of livestock, occurs during the rainy season lasting 4 to 5 months [3]. Forage growth almost stops during the following 7 to 8 months inducing a decrease in dry matter (DM) production and forage nutritive value below the nutritive requirement of grazing animals [3,4]. In semi-arid regions, the standing biomass varies from 1.3 tonne to 2.6 tonnes DM/ha depending to vegetation unit and soil type at the end of the rainy season, whereas pastures are grazed during the rainy season [5]. Natural pastures in the western region of Burkina Faso are poor in fodder legume species [6] and are dominated by some perennial and annual herbaceous species such as *Andropogon gayanus, A. fastigiatus, Loudetia togoensis* [5]. Yet, high proportions of legumes in a pasture are highly desirable for their contribution to a balanced diet through their increase N concentration of crude protein (CP) (percentage N × 6.25), energy, vitamins and mineral contents than grasses [7]. Thus, increasing the presence of fodder legumes in natural pastures would greatly contribute to the improvement of the nutritional value and biomass production of these pastures.

Grazers in natural pastures prefer certain palatable species such as the legumes to other herbaceous species (grasses and forbs). This selective grazing allows a significant improvement of the diet quality and animal performance [8], but it often favours growth of unpalatable species, which, in turn, causes a change in plant community species composition [9,10]. Selective grazing threatens the survival of legumes in heavily stocked tropical pastures. Perennial herbaceous legumes are more susceptible to higher grazing intensity than grasses due to their relatively poor grazing tolerance [11]. Thus, the diverse forms of enrichment of grassland vegetation with fodder legume species such as *Stylosanthes* spp. appear to be an efficient strategy that has a large impact on livestock performance [12].

Plant community composition and structure may be largely influenced by germination and seedling establishment from seeds dispersed via animals through seed input, gap creation and nutrient enrichment [13]. The effect of dung deposition in natural pastures has three main components: (i) a source of colonisers in the form of seeds, (ii) creation of small gaps, as a result of the possible death of the vegetation under the dung with potential favourable conditions for germination and seedling growth [[14], [15], [16]] and (iii) local soil nutrient enrichment period. These small created gaps are regeneration sites for seeds dispersed in the dung and for seeds in local soil seed bank or seed rain. Seed dispersal and re-colonisation processes induced by herbivores are thus driving factors that regulate plant community structure and spatial and temporal distribution and viability of plant populations [17]. The colonisation of a plant community by grazers through seed dispersal is influenced by the quality and quantity of browse species present in the pastures and the phenological stage of these browse species (fructification and maturity of fruits/seeds). In semi-arid regions, livestock production is characterized by an extensive pastoral practice based on livestock mobility in space and time in search of grazing and water resources [18].

However, the possibility of the fodder seeds to pass undamaged through the digestive tract of ruminants can be a key factor in determining the ability of seeds of these plants to survive and to be spread in the grasslands and over long distances [19]. Ruminants play a significant role in maintaining the biodiversity of grassland vegetation through spatial and temporal dissemination of viable seeds of fodder legumes, forbs, grasses, shrubs and trees via their faeces [20]. Because of the large areas considered and their remoteness, ruminants themselves could thus be used to enrich grassland vegetation with desirable legume species. This situation is a low cost alternative to spreading fodder legume species over large areas [21,22]. Also, the enrichment of natural pastures with palatable legume species requires the application of phosphorus, because legumes need more phosphorus (P) to maintain their establishment and growth [23]. The deficiency of P causes significant yield reduction in legume crops and decreases number and mass of nodules [23,24].

*Aeschynomene histrix* Poir. and *Stylosanthes hamata* (L.) Taub (Fabaceae) are two potential palatable legumes that could be used to improve natural pastures in semi-arid regions. Both species are drought tolerant, *Striga*-resistant and have high biomass production [25]. As reported by Adjolohoun et al. [26], the DM production ranges from 2 to 8 t ha^−1^ and 1 to 6 t ha^−1^ for *A. histrix* and *S. hamata*, respectively. They also have high nutritive value with crude protein (CP) levels ranging between 17 and 24%, digestibility of the DM between 53 and 70% and low tannin levels.

The value of *A. histrix* and *S. hamata* as targeted species to improve natural pastures has been explored in subtropical zones [27]. However, to our knowledge, little is known about the potential role of legume seed dispersal by ruminants (cattle and sheep) to improve natural pastures in the western region of Burkina Faso. This present study aimed to investigate the potential enrichment of natural pastures by using fodder legume species. Specific objectives were to: (i) assess the biomass and seed productivity of the *A. histrix* and *S. hamata* under two phosphorus fertilization regimes and (ii) evaluate the percentage of seed recovered from cattle and sheep faeces after ingestion of seeds of the both legumes. This paper concludes by pointing to actionable implications for the quantitative and qualitative improvement of the natural pastures in semi-arid zones.

## 2 Material and methods

### 2.1 Study site description

**Fig. 1:**
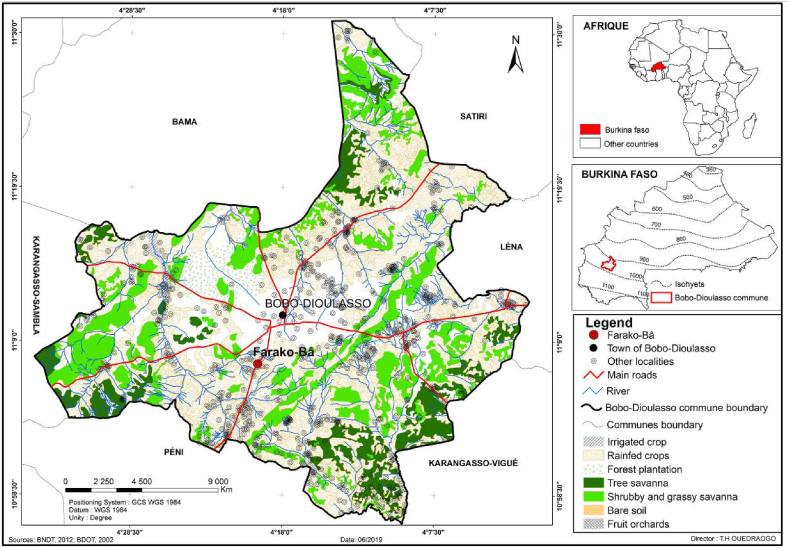
Location of study site.

The experiment was carried out at the Institute of Environment and Agricultural Research located at Farako-bâ (11°06 N, 04°20 W, 405 m. a.s.l., Fig. 1). Phytogeographically, the study site is situated in the southern soudanian zone of Burkina Faso with a mean annual precipitation varying between 900 mm and 1000 mm [28]. The site has a unimodal rainy season, which lasts for about 7 months each year from May to November. The mean annual rainfall for the last decades was 1272 ± 124 mm, and the number of rainy days per annum was 71 ± 6. Mean daily minimum and maximum temperatures ranged from 14 °C to 32 °C in January (the coldest month) and from 25 °C to 41 °C in April (the hottest month). Soil types are mostly the tropical ferruginous to ferrallitic [29]. The soils are characterized by a sandy-loan texture with pH 5.2 to 5.4, average organic matter content of 0.95 to 1.03% and low phosphorus content (41 to 85 mg/kg) [30]. The natural pastures are dominated by woody species such as *Danielia olivieri*, *Afzelia africana, Isoberlia doka, Pterocarpus erinaceus, Prosopis africana, Parkia biglobosa, Burkea africana* and *Albizzia chevalier* and some characteristic herbaceous species such as *Andropogon ascinodis, Anndropogon gayanus, Aristida kerstingii, Ctenium newtonii, Loudetia togoensis, Monocymbium cereciforme, Pennisetum pedicellatum Schizachyrium sanguneum,* etc. Legumes species are rare in the vegetation of the study site. However, the legume *Indigofera* sp., which is mostly non-palatable, occasionally has invaded some of these natural pastures. Aside from agriculture, which is the occupation of 80% of the area's active population, livestock breeding is the most important activity for the generation of household income. The livestock in the study area are comprised mainly by the sheep and cattle.

### 2.2 Plant and animal materials

Seeds of *A. histrix* and *S. hamata* were used in the experiments. The pods of both legumes were collected at seed maturity stage from experimental plots at Institute of Environment and Agricultural Research in Farako-bâ where the species have been growing since 1998. Seeds of *A. histrix* were hand harvested from standing plants, sun-dried and processed to get pure pods as seeds. For *S. hamata*, standing plants were cut carefully and sun-dried, after which the ground was swept to pick up the fallen pods that were processed to get pure pods as seeds. Pods of the two legumes were stored in a cool and dry place prior to initiation of the experiments.

The impact of herbivores on dispersal and recruitment may depend on herbivore size or species [31]. Sheep (small herbivore) may be efficient vectors for seed dispersal in the pastures than cattle due to their numerous pellets while cattle (large herbivore) which consume large amounts of and move over larger distances, may be more effective vectors of seeds over the landscapes [32]. Deposition of faeces in pastures or rangelands should create local fertility favourable conditions to seedlings establishment and development [33,34]. These reasons justified the choice of cattle and sheep in our experiments.

### 2.3 Evaluation of legume biomass and seed production

**Fig. 2:**
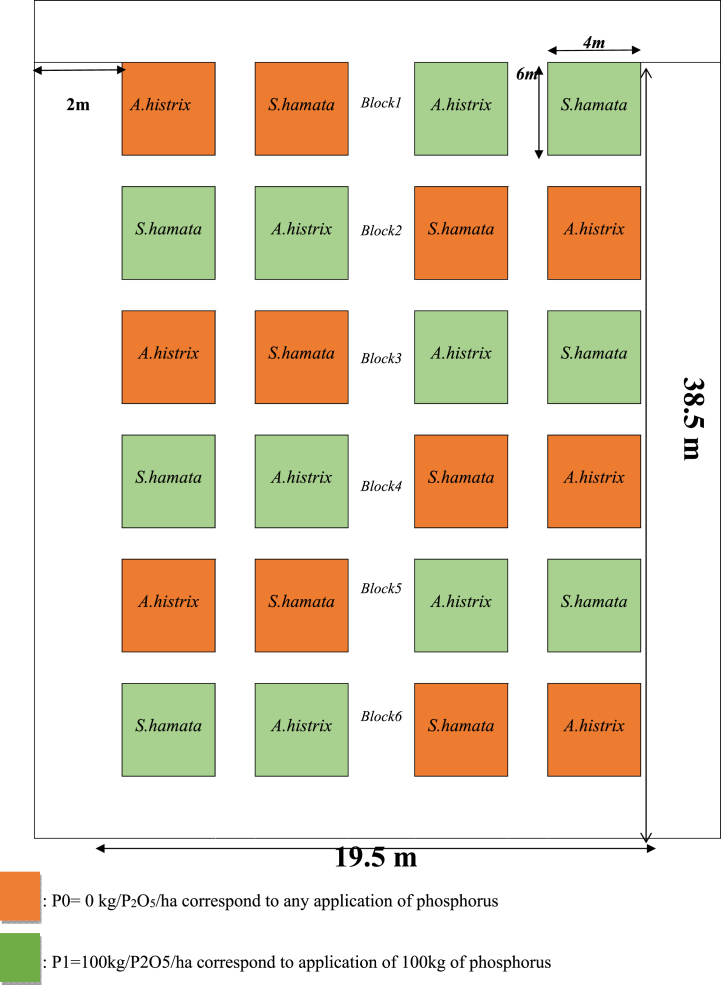
Experimental design.

The seeds of *A. histrix* and *S. hamata* were sowed in a ploughed field according to a complete block design with six replications per treatment making a total of 24 plots that were 6 m × 4 m in area (Fig. 2). The treatments differed in legume species (*A. histrix* and *S. hamata*) and phosphorus application regimes (0 and 100 kg P_2_O_5_/ha/year). For the seed harvest, every experimental unit was divided in two parts for annual seed harvest. For the evaluation of seed production plants of both species were cut at ground level and subsequently the ground was swept to harvest the fallen seeds. Harvested materials were dried separately, hand sieved, and then further cleaned to obtain pure seed samples. Above ground biomass samples were evaluated by hand harvest at ground level in two subplots (1 m^2^) in each plot. Fresh biomass of plants was immediately weighted, labelled, bagged and transported to the laboratory. All the fresh biomass of the same treatment was pooled, and 1 kg sample was taken for oven drying at 65 °C for 48 h to determine dry matter content (DM). For root biomass evaluation, two cubes of 30 cm × 30 cm × 30 cm were taken at the same location where the above ground biomass was evaluated. The soil cores were conditioned in separated bags and hand washed in basins filled with water. Fresh root biomass was dried in an oven at 65 °C for 48 h to determine DM content and get root biomass samples for laboratory analysis.

### 2.4 Seed ingestion method

Four cattle (Zebu) and four sheep (Djallonke) in good health were used to represent the predominance of both herbivores in the study zone [34]. Moreover, it is generally allowed that the variation of the size of the oral cavity and the digestive tract as well as the size and the composition of the faeces can have an effect on the rate of passage and germination of introduced seeds [35]. The sheep used in this experiment were aged 18–20 months with an average live weight of 21 ± 2 kg. The cattle were aged 3–4 years with an average live weight of 202 ± 20 kg. The pods of *A. histrix* and *S. hamata* were fed to the animals mixed with cotton seed cake. Incorporation rate was calculated on the basis of 5% of the ingestion capacity that was approximately 40 g/sheep and 219 to 315 g/cattle per day. The pods of the both legumes were previously sieved manually at 0.1 mm meshes to facilitate their recovery in deposit. The crushed cotton seed cake was used on the basis of 20% of ingestion capacity that was 200 g/day for sheep and from 870 to 1270 g/day for cattle. The mixture was given to animals only once a day in the morning between 08:00 a.m. and 09:00 a.m. During the ration distribution, we ensured that the mixture was completely ingested by animals. After this, animals were supplemented with hay of *Pennisetum pedicellatum* harvested at the Farako-Bâ research station at the flowering initiation stage. Water was distributed ad-libitum. Animals were weighed at the beginning and the end of the experimentation.

### 2.5 Seed recovery

The collection of deposit began 24 h after ingestion of the distributed ration described above. For the sheep, it was made using digestibility breeches. For bovines, deposits were collected on the ground, each animal was isolated in a well-cleaned pen for the circumstance. The 24 h of waiting correspond to the average retention time of the digesta in the rumen depending on feeding level and environmental conditions particularly temperature [36]. Gokbulak and Call [37] found that the time corresponding to maximum flow of seeds through the digestive systems was 24 and 48 h after ingestion. After defecation, 100 g sample of faeces was collected per animal for seed search. The moist cattle faeces were mixed by hand in a bucket of water. The sheep faeces, which were dry were carefully crushed by hand to facilitate mixing with water. The faeces were filtered through a sieve of 1 mm of mesh. The residues obtained were air-dried. Seeds were sorted, removed by hand after drying.

### 2.6 Germination tests

Seed germination tests were conducted in laboratory and greenhouse.

#### 2.6.1 Germination test in laboratory

Three types of seeds were used in germination tests in the laboratory: seeds collected from sheep pellets and cattle dung and a nontreated control. Seeds collected from pellets or dung were divided into lots of 100 seeds per animal and there were four replications of 100 seeds in each case. Seeds were placed in Petri dishes on two layers of filter papers moistened with distilled water [31,38]. Germination tests were carried out in a growth chamber at a constant temperature of 32 °C in light provided by a fluorescent lamp (F40 W/33 RS cool white light) placed below the vat [31,39]. The experiment was run for 21 days for all sources of seeds. Germination was monitored on daily basis, and seeds with a radicle 2 mm in length were counted and considered as seedlings germinated and discarded [38,40].

#### 2.6.2 Germination in greenhouse

The test concerned faeces collected 24 h after seed ingestion. Four samples (50 g each) of fresh faeces were collected from sheep and from cattle and placed on the soil surface in buckets (12 litters) filled of sterilized soil. Faeces were slightly covered with the soil to support humidification. The buckers then were placed in a greenhouse and were sprinkled twice per day with enough water to reach holding capacity while avoiding water flow. The bottom of the buckets was pierced with 3 holes to allow excess water percolation. Germinated seeds from all tests were counted every week and seedlings discarded [33,41]. The seeds were incubated at the greenhouse for 21 days.

### 2.7 Calculation and statistical analysis

The parameters were the number of seeds in faeces per day, the percentage of seeds recovered compared to the quantity fed to animals, the germination percentage of seed recovered from faeces, the speed and the average time of germination of seeds in faeces of cattle and sheep were described using the following formulas.

$$Content\;of\;seeds=\frac{Seed\;re\text{cov}ered\;from\;faeces\;sample\;weight}{Faeces\;sample\;weight\;DM}\times 100$$

$$Percentage\;of\;recovery=\frac{Seed\;rate\;in\;faeces\;sample\times Total\;faeces\;DM\;weight}{Seed\;daily\;ingested\;weight}\times 100$$

Velocity coefficient: $\frac{\sum }{\sum }$.

Mean time of germination:

$$MGT=\frac{1}{CV}\times 100$$

Where N_i_ = number of germinated seeds day i and, J_i_ = number of days after sowing (the first day of sowing not being considered).

To analyse the effect of passage through the cattle and sheep on germination, percentage of germination were arcsine transformed to improve homogeneity of variance and normality before analysis [42,43]. The mean biomass productivity of the both legumes according to the phosphorus application regime and year was calculated. When significant differences were detected between treatments, a pair-wise comparison was made using Tukey's Honestly Significant Difference test at 5% level of significance.

To compare cattle and sheep in the mean number of germinated seedlings of *A. histrix* and *S. hamata* in faeces of cattle and sheep, parametric tests (paired t tests) were used. Further differences between cattle and sheep in the dispersal of seedlings of both legumes were analyzed using generalized linear models (GLMs). The response variable in the models was the number of seedlings. All GLMs were carried out using the ‘lme4’ package with Poisson distribution as the data originated from counts [41,44]. We tested the data for over-dispersion, and if so, we used a negative binomial distribution. All models were checked for homogeneity of variance. Data were presented as means ± standard error. Results were considered significant when P˂0.05. All analyses were performed using the statistical software R [45].

## 3 Results

### 3.1 Seed and biomass production of both legume species

**Fig. 3:**
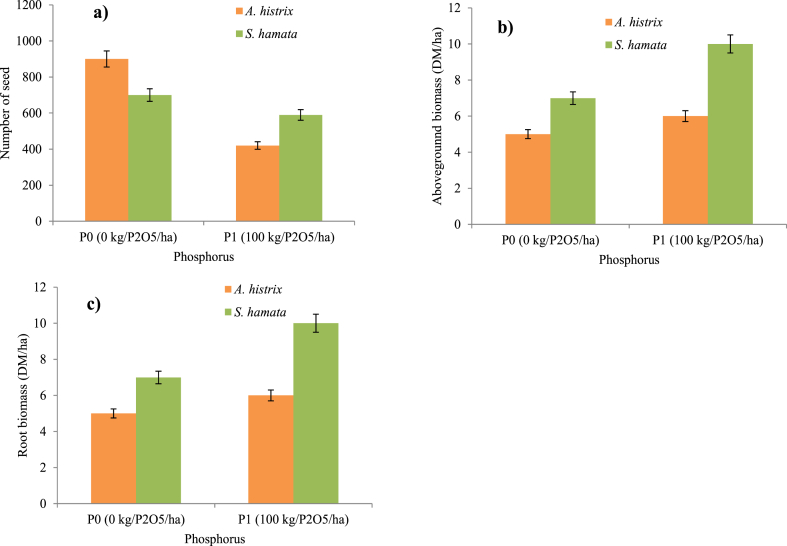
Effect of phosphorus applications (P0 = 0 kg/ha of P_2_O_5_ and P1 = 100 kg/ha of P_2_O_5_) on seed and biomass production of *A. histrix* and *S. hamata.* Means ± SE with different letters are significantly (P < 0.05) different based on Tukey's HSD test.

Seed production of *A. histrix* was higher than that of *S. hamata* either with or without phosphorus application (P˂0.05, Fig. 3). Thus, during four years of study, the average number of seeds of *A. histrix* was 909 ± 358 and 687 ± 245) and that of *S. hamata* was 441 ± 133 and 496 ± 151) with or without phosphorus application, respectively (Fig. 3a). Seed production of *A. histrix* and *S. hamata* increased in the second year with and without phosphorus application) but decreased during the two last years (Fig. 3). Aboveground biomass of *S. hamata* was higher than that of *A. histrix* either with or without phosphorus application (Fig. 3b). Also, the root biomass of *A. histrix* was higher than that of *S. hamata* either with or without phosphorus application (Fig. 3b). GLM result showed that species and year of harvest had a significant effect on seed yield (P < 0.0001). The interaction between legume species and phosphorus application regime had a significant effect on legume seed yield (P = 0.001, Table 1). Species response to phosphorus application is not the same for *A. histrix* and *S. hamata*. We observed that the yield of legume pods was higher in the second year than in the other years. After this year, pod yield decreased slightly for both legumes with or without phosphorus application. For *A. histrix*, phosphorus application had a significant depressive effect on seed yield (P0>P1). Year of harvest also had a significant effect on seed yield. With *S. hamata*, the same result but phosphorus application increased seed yield (P0<P1). Year of harvest also had a significant effect on seed yield but not the interaction between phosphorus application regime and year of harvest.

**Table 1:** Results of the Generalized Linear Model (GLM) analysis of seed production of *A. histrix* and *S. hamata*.

Source of variation	DF	F	P
Year (Y)	3	9.548	**< 0.00001**
Species (S)	1	42.680	**< 0.00001**
Phosphorus (P)	1	1.440	0.234
Y × S	3	1.920	0.133
Y × P	3	0.017	0.997
S × P	1	12.953	**0.00101**
Y × L × P	3	0.457	0.713

Note: SD: Df: Degree of freedom; F: variation between samples means or variation within the samples; P: value of probability. The values of probability in bold face are significant (P˂0.05).

Analysis of variance by GLM of the effects of the factors “legume species” and “phosphorus application” on the above-ground and root biomass showed that legume species and phosphorus application had a significant effect on above-ground, root and total biomass yield (P˂0.05). The interaction between the two factors had no significant effect on yield whereas this interaction had a significant effect on the sum of the above ground and root biomass for the both legumes (Table 2).

**Table 2:** Results of the Generalized Linear Model (GLM) analysis of biomass yield (t DM/ha) of *A. histrix* and *S. hamata*.

Parameters	Sources of variation	Df	F	P
Above-ground biomass	Intercept	1	514.123	
	Species (S)	1	10.986	**0.003**
	Phosphorus (P)	1	32.074	**<0.00001**
	S × P	1	2.915	0.103
Root biomass	Intercept	1	156.144	
	Species (S)	1	7.902	**0.011**
	Phosphorus (P)	1	0.144	0.709
	S × P	1	0.716	0.408
Total biomass	Intercept	1	570.485	
	Species (S)	1	0.011	**<0.00001**
	Phosphorus (P)	1	0.709	**<0.0001**
	S × P	1	0.408	**0.044**

Total biomass is the sum of aerial and root biomass of *A. histrix* and *S. hamata*.

The values of probability in bold face are statistically significant (P˂0.05).

### 3.2 Seed recovery and germination

Seed recovery percentage was higher with cattle compared to sheep for the same plant species (P˂0.05). For cattle, seed recovery percentage was higher for *A. histrix* than *S. hamata* (Table 3). For sheep, seed recovery did not differ significantly for *A. histrix* and *S. hamata* (P > 0.05). For *A. histrix,* recovered seeds from either cattle and sheep dung showed a significantly lower percentage of germination compared to control seeds in the order sheep < cattle « control (Table 3).

**Table 3:** Effect of animal (cattle and sheep) and legume species on seeds recovery *(A. histrix.* and *S. hamata*) from cattle and sheep faeces. Germination and mean time of germination (MTG) are compared to control seeds.

Animal	Legumes	Seed recovery (%)	Germination (%)	MTG (days)
Cattle	*A. histrix*	17.95^a^	12.68^d^	3.00 ^b^
	*S. hamata*	9.35 ^b^	27.50^c^	2.25^c^
Sheep	*A. histrix*	0.85^c^	44.38 ^b^	3.75 ^b^
	*S. hamata*	1.00^c^	44.50 ^b^	3.25 ^b^
Control	*A. histrix*		93.25^a^	3.25 ^b^
	*S. hamata*		**7.75**^**d**^	**3.75**^**b**^

The mean percentages with different letters are significantly different based on Tukey test at 5% level.

For *S. hamata*, seeds recovered from sheep dung had a significantly higher germination percentage than those of control (seeds of both legume species collected at the field). For this species, seed germination percentage was control < cattle < sheep. The germination percentage of *A. histrix* and *S. hamata* seeds recovered from sheep dung was higher than those of seeds recovered from cattle dung.

For *A. histrix*, no significant difference was observed for mean time of germination (MTG) for seeds collected from dung of cattle, sheep and control. For *S. hamata*, seeds recovered from dung of cattle and sheep had a MTG less than the control. Values are ordered as control < sheep < cattle (Table 3).

### 3.3 Seed germinating directly in faeces in greenhouse

Seed content in cattle dung was significantly higher for *A. histrix* than for *S. hamata,* and, the number of sprouting seeds was significantly higher for *A. histrix* and less for *S. hamata* (Table 4).

**Table 4:** Effect of type of animal (cattle and sheep) and legume species *(A. histrix* and *S. hamata*) on total *s*eed number and number of seeds sprouted in 100g DM faeces of cattle and sheep in the greenhouse.

Animal	Legumes	Total seed number in 100 g DM faeces	Sprouting seed number in 100 g DM faeces
Cattle	*A. histrix*	2296^a^	292^a^
	*S. hamata*	487 ^b^	132 ^b^
Sheep	*A. histrix*	70^c^	31^c^
	*S. hamata*	78^c^	36^c^

Means in a row followed by the different letter are significantly different (P˂0.05).

DM: dry matter.

In case of sheep, there was no significant difference between total numbers of *A. histrix* and *S. hamata* seeds in faeces. Consequently, there was no difference between number of sprouting seeds of the two legumes in sheep faeces.

## 4 Discussion

### 4.1 Seed and biomass production by both legume species

The seed yield production of *A. histrix* and *S. hamata* was variable depending on phosphorus application. Phosphorus application decreased seed yield of *A. histrix* but increased seed yield of *S. hamata*. The seed yield of 496 kg/ha observed with *S. hamata* was comparable to that obtained by Norton et al. [46], in which a seed yield of 483 and 903 kg/ha was for standing plants cut at 2 cm above soil and for fallen seeds collected with vacuum cleaner, respectively, in similar climatic conditions as in our case. This difference can be attributed to harvest techniques employed by Norton et al. [46] who concluded that the suction harvesting is more preferable to direct heading for collecting the most seed yield. Phosphorus application increased significantly the above ground and root biomass production of both legume species as reported by several authors [23,24]. The biomass observed in our study with *A. histrix* is similar to that obtained by Nworgu and Ajayi [47], which ranged from 6.1 to 7.25 t DM/ha with 60 kg P_2_O_5_/ha application in Western Nigeria to 3.9 t DM/ha obtained in pure culture in the guinea savannah of Côte d’Ivoire. The high biomass found by phosphorus application augurs the available crude protein for livestock during the year.

The findings of our study are in agreement with Adjolohoun [48] who argued that the combination of these two forage groups can satisfy the nutrients needs of ruminants.

### 4.2 Seed recovery

Seed recovery was higher for cattle (17.95% and 9.35% for *A. histrix* and *S. hamata*, respectively) than for sheep (0.85% and 1% respectively, for the same species, suggesting that economically, cattle are better seed dispersers of the two legumes than sheep. This is in accord with Zhang et al. (2013) who counted in a similar trial with seeds of *Zoysia japonica,* 408.80 ± 18.01 equivalent to 81760.00 ± 3601.67 seeds/kg in cattle dung, while sheep dung contained 165.73 ± 17.23 intact seeds, equivalent to 33146.67 ± 3446.76 seeds/kg.

Seed recovery varied between the two legumes species of this study based on seed mass. For example, *A. histrix* seeds were smaller (1.8 mg seed^−1^) than *S. hamata* seeds (2.4 mg seed^−1^). Ghassaly et al. [49] in similar research observed that more small seeds (0.5–1.5 mg/seed) were recovered (59–72%) than large seeds (2.5–4.9 mg/seed) at 10–40%. Ghassali et al. [49] have observed that the 0.45 mg seeds of *Trifolium campestre* disintegrated less (72% passage) than the 2.68 mg of *T. haussknechtii* (10% passage). Sanou et al. [33] also reported that seeds with a small mass are dispersed by ruminants.

Whitacre et al. [50] have observed that the differences in physical seed properties (size, mass, shape and seed coat) influenced interspecies variation in seed recovery with cattle. Diet quality also has been demonstrated to influence the amount of seeds recovery with sheep [51]. These authors observed that with a low-quality diet, only 10% of the seeds were recovered, whereas with medium and high-quality diets 28% of the seeds were recorded. Many studies have found that significantly more seeds are recovered from cattle than sheep dung [19,31,35]. However, *A. histrix* seeds recovered were mainly still in pods and a minor part was without pods. In contrast, *S. hamata* seeds recovered were essentially without pods. This is in line with Simao and Jones [52], who observed the same situation with *S. seca*. These authors argued that amount of seeds recovered while remaining in pods depends on diet quality, with a low-quality diet having up to 6% and medium and high quality diets showing 11% seed recovery.

### 4.3 Seed germination

The germination percentage of *A. histrix* seeds was significantly lower than that of control, particularly with seed recovered from cattle dung. We suspected that this result was due to germination initiation in the faeces shortly after defecation, since cattle dung contains a high amount of water. In practice, dung was collected every 24 h, but some seeds in faeces deposited earlier in the 24-h period may have germinated. This is probable since we observed that a high percentage of *A. histrix* seeds germinated within 24 h after germination tests were initiated.

Since sheep pellets contain little water, the seeds were less likely to germinate than those in cattle dung. This explains why germination of seeds recovered from sheep pellets was higher compared to those recovered in cattle dung. Indeed, endozoochorous seed dispersed involves consumption by a herbivore and thus exposure to different kinds of digestives fluids during passage through the gastrointestinal tract [53]. For *S. hamata*, seeds recovered from cattle dung germinated better than control. The passage of legume seeds through the gut of ruminants could break their dormancy and thereby lead to an increased percentage of germination [31,35]. This situation is linked by the fact that the survival of seed passing through the ruminant digestive tract is influenced by residence time and exposure to digestive enzymes [10]. Thus, this would explain why more seeds of *S. hamata* than *A. histrix* were recovered during our experiment. Also, several studies on evaluation of soil seed bank in grazed sites showed that the grazed sites had a high seed density depending on the level of grazing intensity [33].

For *A. histrix* and *S. hamata*, the germination percentage is higher in sheep pellets than in cattle dung. This result corroborated the findings of Simao and Jones [54] who stated that the viability of seeds within faecal pellets of sheep and goats was less affected than that in cattle dung due to their lower moisture content [55]. In another experiment, Simao et al. (1987) observed that cattle digested less seeds than did sheep and goats, but the germination characteristics of the recovered seeds were similar for different animals. Seeds of both study species differed in germination characteristics before and after passage through the gut of ruminants. Thus, seeds of both legumes are tolerant of endozoochoory. Also, our results corroborated with those of Peco et al. [56] who observed differences in seed germination of 20 abundant species from central Iberian rangelands. The germination percentage was higher in control for some (75%) and less for other species compared to seeds treated with simulated herbivore consumption.

Transportation, dissemination and the germination of seeds of the desired legumes are important for natural pastures improvement. The second aspect we have to deal with will be their lack of persistence.

## 5 Conclusion

This study sought to provide important information on the effects of cattle and sheep on legume seed dispersal and the role of phosphorus in biomass and seed production of legumes to formulate strategies that could contribute to the enrichment of natural pastures of semi-arid zones. Results showed that the targeted legumes have high seed and biomass production capacities. The mean numbers of seeds that germinated from the cattle dung were significantly higher than those from sheep pellets (P˂0.05). Thus, cattle are better animals for seed dispersal into pastures than sheep. Seeds recovered from all dungs germinated quickly. This is a major advantage that could facilitate the growth and establishment of seedling populations of these legume species in pastures. These findings can encourage pastoralists to enrich natural pastures in semi-arid zones by spreading manure rich in legume seeds, planting or sowing seeds of legumes species.

## Author contribution statement

Lassina Sanou, Souleymane Ouédraogo: Conceived and designed the experiments; Analyzed and interpreted the data and Wrote the paper.

Patrice Savadogo, Chantal Yvette Kaboré-Zoungrana and Jerome Bindelle: Performed the experiments; Interpreted the data and Wrote the paper.

## Data availability statement

Data will be made available on request.

## Additional information

No additional information is available for this paper.

## Declaration of competing interest

The authors declare that they have no known competing financial interests or personal relationships that could have appeared to influence the work reported in this paper.
